# Copper Hexacyanoferrates Obtained via Flavocytochrome *b*_2_ Assistance: Characterization and Application

**DOI:** 10.3390/bios15030157

**Published:** 2025-03-02

**Authors:** Galina Gayda, Olha Demkiv, Nataliya Stasyuk, Halyna Klepach, Roman Serkiz, Faina Nakonechny, Mykhailo Gonchar, Marina Nisnevitch

**Affiliations:** 1 Department of Analytical Biotechnology, Institute of Cell Biology National Academy of Sciences of Ukraine, 14/16 Drahomanov Str., 79005 Lviv, Ukraine; 2 Department of Biology and Natural Sciences, Drohobych Ivan Franko State Pedagogical University, 82100 Drohobych, Ukraine; 3 Department of Chemical Engineering, Ariel University, Kyriat-ha-Mada, Ariel 4070000, Israel

**Keywords:** green synthesis of copper hexacyanoferrate, yeast flavocytochrome *b*_2_, multi-functional sensor, amperometric and optic biosensors, fluorescence, perovskite

## Abstract

Artificial enzymes or nanozymes (NZs) are gaining significant attention in biotechnology due to their stability and cost-effectiveness. NZs can offer several advantages over natural enzymes, such as enhanced stability under harsh conditions, longer shelf life, and reduced production costs. The booming interest in NZs is likely to continue as their potential applications expand. In our previous studies, we reported the “green” synthesis of copper hexacyanoferrate (gCuHCF) using the oxidoreductase flavocytochrome *b*_2_ (Fc*b*_2_). Organic–inorganic micro-nanoparticles were characterized in detail, including their structure, composition, catalytic activity, and electron-mediator properties. An SEM analysis revealed that gCuHCF possesses a flower-like structure well-suited for concentrating and stabilizing Fc*b*_2_. As an effective peroxidase (PO) mimic, gCuHCF has been successfully employed for H_2_O_2_ detection in amperometric sensors and in several oxidase-based biosensors. In the current study, we demonstrated the uniqueness of gCuHCF that lies in its multifunctionality, serving as a PO mimic, a chemosensor for ammonium ions, a biosensor for L-lactate, and exhibiting perovskite-like properties. This exceptional ability of gCuHCF to enhance fluorescence under blue light irradiation is being reported for the first time. Using gCuHCF as a PO-like NZ, novel oxidase-based sensors were developed, including an optical biosensor for L-arginine analysis and electrochemical biosensors for methanol and glycerol determination. Thus, gCuHCF, synthesized via Fc*b*_2_, presents a promising platform for the development of amperometric and optical biosensors, bioreactors, biofuel cells, solar cells, and other advanced devices. The innovative approach of utilizing biocatalysts for nanoparticle synthesis highlights a groundbreaking direction in materials science and biotechnology.

## 1. Introduction

Nanozymes as effective mimics of natural enzymes are promising for applications in various fields of biotechnology. The development of simple methods for nanozyme synthesis and characterization and the exploration of new application areas are currently emerging challenges [1,2].

Hexacyanoferrates of transition metals (HCFs) are cost-effective materials that can be synthesized using various techniques, including chemical methods and environmentally friendly approaches known as “green synthesis”. HCFs have potential applications in a wide range of fields, including fundamental research, environmental science, medicine, and industry [3,4,5]. Due to their redox activity, super-magnetic properties, and nanoscale size, HCFs are widely used in electrochemistry [5,6,7,8], optics [9], molecular magnetism [10], bioreactors, and as cathode materials for (bio)fuel cells [3,11,12,13,14,15,16,17,18]. As artificial peroxidase (PO), HCFs are employed in optical and amperometric biosensors [19,20,21]. Despite their multifunctionality, the composition of HCFs is complex and largely influenced by synthesis methods and storage conditions [3,6,7,17,18,20].

In our previous research, we demonstrated the feasibility of developing reagentless amperometric biosensors (ABSs) using HCFs of transition and noble metals synthesized via the yeast oxidoreductase flavocytochrome *b*_2_ (Fc*b*_2_). These green-synthesized metal–organic materials, consisting of HCFs incorporated with the enzyme, were termed gHCFs and extensively characterized for their structure, size, composition, catalytic properties, and electro-mediator activity. Among these, copper hexacyanoferrate (gCuHCF), which emerged as the most effective PO mimic, was subjected to detailed investigation. When immobilized on a graphite electrode, gCuHCF exhibited intrinsic amperometric signals for hydrogen peroxide under specific conditions of pH and working potential. Furthermore, we demonstrated that gCuHCF could serve as a PO-mimic in an amperometric sensor for hydrogen peroxide analysis and as a sensing platform in mono-enzyme ABSs for the determination of glucose [21] and arginine [22]. Additionally, due to its high electroactivity, gCuHCF was successfully applied as a mediator of electron transfer in laccase- and Fc*b*_2_-based ABSs for the analysis of catechol [23] and lactate [24], respectively. It is worth mentioning that, in all cases, the usage of gCuHCF resulted in increased sensitivity of the developed ABSs.

Various methods were employed to analyze the morphology, structure, and composition of the synthesized gCuHCF. SEM images revealed that the particles exhibited a flower-like shape with diameters in the microscale range, while the petal thickness was in the nanoscale range. The microscale size (3.04 ± 1.98 μm) of gCuHCF was confirmed through particle counting, dynamic light scattering, and zeta potential analysis. All methods indicated that the tested sample is not monodisperse [21]. FTIR analysis confirmed the presence of both organic and inorganic components in the material, specifically identifying copper cyanoferrate particles and organic compounds, likely of protein origin. An X-ray diffraction (XRD) analysis estimated that the gCuHCF possesses a cubic centrosymmetric crystalline structure, with a cell parameter of 7.071 Å [21].

Flower-like micro-nanosized organic–inorganic materials offer several advantages in medicine, biotechnology, bioelectronics, and industry due to their high surface area, biocompatibility, electrical conductivity, catalytic efficiency, and versatility [25,26,27].

Combining natural enzymes with suitable PO-like nanozymes offers a promising strategy for developing enhanced and cost-effective enzymatic methods, including ABS. This approach delivers improved characteristics, such as high stability and increased sensitivity to the target analyte.

In the current study, we present the results of further investigations into gCuHCF, highlighting the multifunctionality of this organic–inorganic material. It functions as a PO mimic, a chemosensor for ammonium ions, a biosensor for L-lactate, and demonstrates notable perovskite-like properties.

## 2. Materials and Methods

### 2.1. Reagents

Inorganic salts, chromogenic substrates, hydrogen peroxide, Nafion, methanol, glycerol, and all other chemicals of analytical grade were supplied by Sigma-Aldrich (Steinheim, Germany). All chemicals were dissolved in ultrapure water from the Milli-Q^®^ IQ 7000 Water System, produced by Merck KGaA (Darmstadt, Germany).

### 2.2. Enzymes

All enzymes were purified according to the methods developed by the authors [22,28,29,30]. Highly purified flavocytochrome *b*_2_ (Fc*b*_2_, EC 1.1.2.3) and alcohol oxidase (AO, EC 1.1.3.13) were isolated from the yeast *Ogataea polymorpha* [28]. Glycerol oxidase (GlycO) was purified from the fungus *Botrytis allii* [29]. Purified L-arginine oxidase (ArgO, EC 1.4.3.25) was obtained from the mushroom *Amanita phalloides* [22].

Activities of AO, ArgO, and GlycO were determined by the rate of hydrogen peroxide formation in reaction with correspondent substrates (methanol, Arg, and glycerol), as monitored by the peroxidase oxidation of *o*-dianisidine (*o*-DZ) in the presence of PO [22,28,29,30]. The optical density of the colored product was determined at 525 nm using a Shimadzu UV1650 PC spectrophotometer (Kyoto, Japan).

### 2.3. Synthesis and Characterization of CuHCF

Nanoparticles of CuHCFs, obtained via enzyme-mediated (gCuHCF) and chemical (chCuHCF) methods [21], were collected by centrifugation, and the precipitates were rinsed twice with water and stored as a water–colloid solution at +4 °C until use.

Pseudo-peroxidase (PO-like) activity of the gCuHCF was measured using the colorimetric methods with o-DZ and ABTS as chromogenic substrates in the presence of H_2_O_2_ [21].

### 2.4. Apparatus and Measurements

The structure and morphology of gCuHCF were examined using a REMMA-102-02 SEM microanalyzer (Selmi, Sumy, Ukraine) [21]. The fluorescence properties of gCuHCF were analyzed using an inverted fluorescence microscope (Axio Lab. A1, Carl Zeiss, Oberkochen, Germany) and a Shimadzu RF-6000 spectrofluorophotometer (Kyoto, Japan).

Amperometric experiments were conducted as described in our previous paper [21], using a Pt wire as the counter electrode, an Ag/AgCl/3M KCl electrode as the reference, and a graphite rod with a diameter of 3.05 mm as the working electrode (GE). The development and characterization of amperometric sensors and enzyme-based biosensors, along with the statistical analysis of measurements, have been conducted as previously described [21,22,23,24,28].

## 3. Results and Discussion

### 3.1. The gCuHCF as PO Mimic in the Photometric Method for H_2_O_2_ Detection

**Figure 1:**
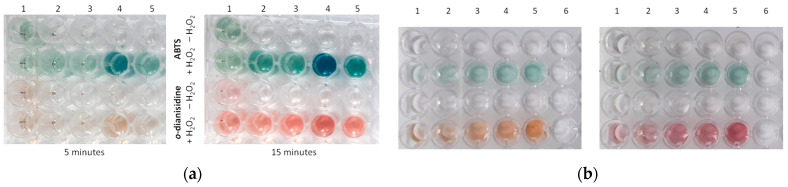
Examples of visualization in the ABTS- and *o*-dianisidine-based assays: (**a**)—PO-like activity of the chCuHCF (1) and gCuHCF (2–5) samples with the following activities (U/mL): 1—1.38; 2—2.12; 3—1.98; 4—3.78; 5—2.44. The substrate for PO-like activity contains a constant H_2_O_2_ concentration. (**b**)—The dependence of color intensity on increasing H_2_O_2_ concentrations (1–5), compared to the control sample without H_2_O_2_ (6).

In our previous work [21], we characterized the morphology, structure, and composition of gCuHCF in detail (Figure S1) and demonstrated the high specificity of this organic–inorganic hybrid material for H_2_O_2_ (Figure S2). The gCuHCF was applied as a chemosensor in the development of a photometric method for H_2_O_2_ detection. During the initial stage, the most effective CuHCF samples were selected from the green and chemically synthesized variants (gCuHCF and chHCF, respectively). For this purpose, naked-eye colorimetric screening of various samples was conducted to assess their PO-like and nonspecific laccase-like activities (Figure 1a). The analysis revealed that chCuHCF exhibits both activities, whereas gCuHCFs display only PO-like properties. The gCuHCF sample #4, which exhibited the highest PO-like activity, was selected for the development of colorimetric tests for H_2_O_2_. Figure 1b illustrates the visualization of a semi-quantitative screening test for rapid and specific detection of H_2_O_2_ in a microplate.

**Figure 2:**
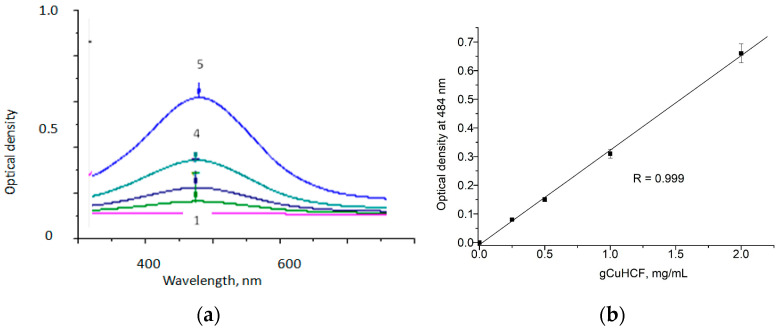
Optical spectra of gCuHCF solutions (**a**) at different concentrations (mg/mL): 0 (1), 0.25 (2), 0.5 (3), 1 (4), 2 (5), and the calibration graph for photometric gCuHCF determination (**b**).

To establish a quantitative analysis method for H_2_O_2_ and determine the kinetic characteristics of gCuHCF, we developed a photometric approach for accurately measuring its concentration. For this purpose, we analyzed the spectra of gCuHCF solutions with varying concentrations and identified the optical density at 484 nm for each sample (Figure 2a). This allowed us to establish a correlation between the concentration of gCuHCF and the measured optical density (Figure 2b).

**Figure 3:**
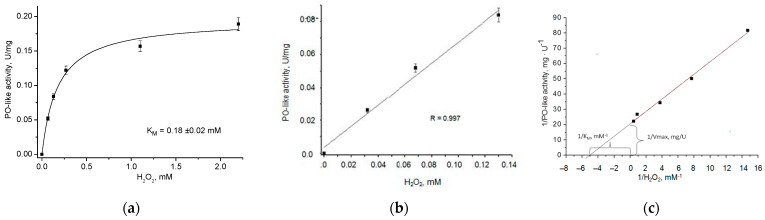
Dependence of the optical density of the reaction mixture on H_2_O_2_ concentration (**a**,**b**) and kinetic data linearization using the Lineweaver–Burke method (**c**). The initial gCuHCF concentration is 4.2 mg/mL.

The results of the development of the gCuHCF/*o*-DZ-based method for quantitative H_2_O_2_ analysis are presented in Figure 3. This figure illustrates the calibration graph (Figure 3a) and linearity (Figure 3b) in detecting H_2_O_2_.

The analysis of results in Figure 3 enabled the determination of the kinetic constants of gCuHCF with respect to H_2_O_2_. The Michaelis–Menten constant (K_M_) was found to be 0.2 mM, and the maximum reaction velocity (V_max_) was 0.05 μmol·min^−1^·mg^−1^. A comparison of the K_M_ values of gCuHCF and natural PO (3.7 mM as reported in [20]) demonstrates the advantage of nanoPO, as gCuHCF exhibits a lower K_M_, signifying a higher affinity for H_2_O_2_ than natural enzymes. According to Figure 3b, the linear range for H_2_O_2_ determination extends up to 0.13 mM, with a limit of detection (LOD) of 10 µM. This suggests that gCuHCF is a promising artificial PO in oxidase-peroxidase optical methods for analyte determination when paired with appropriate oxidases.

The specificity of gCuHCF to H_2_O_2_ is a highly valuable characteristic, making it suitable for use as a selective PO-mimetic element in amperometric- and colorimetric-oxidase-based biosensors. The main principle of the colorimetric method was described for alcohol oxidase (AO), natural PO, and various chromogenic chemicals [30].

**Figure 4:**
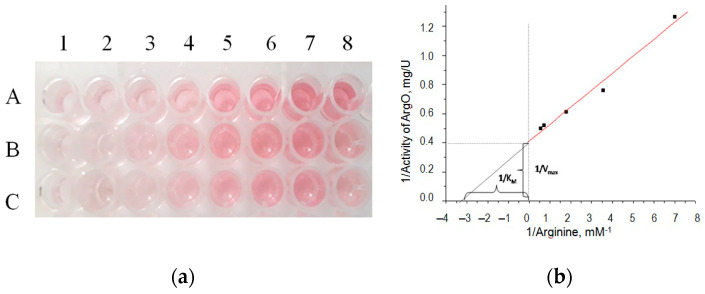
Examples of calibration graphs in the ArgO/gCuHCF/*o*-DZ naked-eye method for Arg determination (**a**), and the linearization of kinetic data using the Lineweaver–Burke method (**b**). The reaction mixtures (**a**) contain increasing concentrations of Arg (mM): 0 (1), 2 (2), 5 (3), 10 (4), 25 (5), 50 (6), 75 (7), 100 (8), with ArgO (1 U/mL) and varying gCuHCF concentrations (mg/mL): 4 (A), 2 (B), and 1 (C).

Examples of the applicability of the gCuHCF/*o*-DZ method for arginine (Arg) analysis are presented in Figure 4. We used arginine oxidase (ArgO) for the naked-eye approach (Figure 4a) and in the quantitative determination of kinetic constants of the enzyme with respect to Arg (Figure 4b). These findings demonstrate the potential effectiveness of the gCuHCF/*o*-DZ method for quantitative Arg analysis, highlighting its applicability in optic biosensing and offering a reliable approach for detecting and measuring Arg concentrations.

The analysis of results in Figure 4 enabled the determination of the kinetic constants of ArgO in the ArgO/gCuHCF/*o*-DZ method with respect to Arg. The K_M_ was found to be 0.3 mM, and the V_max_ was 2.5 μmol·min^−1^·mg^−1^.

### 3.2. Application of gCuHCF as a PO Mimic in Amperometric Biosensors

In our previous papers, we demonstrated the applicability of gCuHCF as a chemosensor for amperometric H_2_O_2_ detection, using a commercial disinfectant as a real sample [21]. Additionally, a highly sensitive mono-enzyme ABS was developed using commercial glucose oxidase (GO) and gCuHCF as an artificial PO. This proposed ABS was successfully tested on commercial juice samples for glucose analysis [21].

The obtained results suggested that gCuHCF may be a promising micro-nanomaterial for the construction of ABSs with any oxidase. As an electroactive, cost-effective composite with intrinsic PO-like activity, gCuHCF could be useful in the development of practically important and economically efficient ABSs.

We proposed gCuHCF-based ABSs with enhanced analytical characteristics for the Arg analysis, using ArgO [22], for catechol analysis, using laccase [23], and for L-lactate analysis, using Fc*b*_2_ [24].

**Figure 5:**
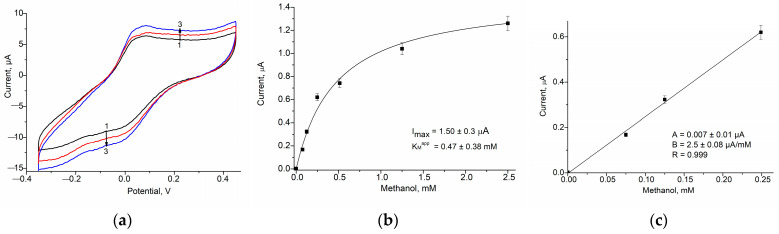
Characteristics of the AO/gCuHCF/GE as an ABS for methanol: The CV profiles as outputs on methanol addition (**a**) up to concentration (mM): 0 (1, black), 2.5 (2, red) and 5 (3, blue), and the dependence of the current response on increasing concentrations of the analyte for a wide range (**b**) and a linear range (**c**). The GE was modified with 20 mU of gCuHCF exhibiting PO-like activity and 200 mU of AO. Conditions: scan rate (for **a**) is 50 mV·s^−1^ vs. Ag/AgCl (reference electrode), working potential (for **b**,**c**) is 150 mV; 50 mM phosphate buffer, pH 7.0, 20 °C.

**Figure 6:**
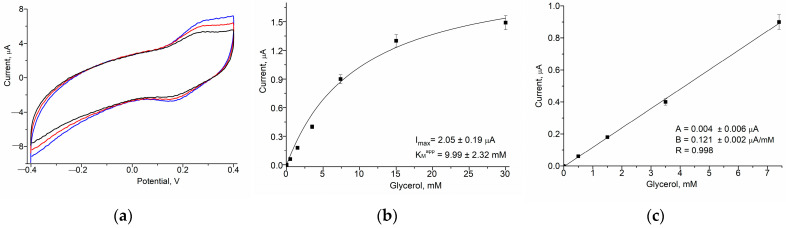
Characteristics of the GlycO/gCuHCF/GE as an ABS for glycerol: The CV profiles as outputs on glycerol addition (**a**) up to concentration (mM): 0 (black), 12 (red), and 24 (blue); and the dependence of the current response on increasing concentrations of the analyte in the wide (**b**) and linear (**c**) ranges. GE was modified with 20 mU of gCuHCF exhibiting PO-like activity and 150 mU of GlycO. Conditions: scan rate (for **a**) is 50 mV s^−1^ vs. Ag/AgCl (reference electrode), working potential (for **b**,**c**) is 150 mV; 50 mM phosphate buffer, pH 8.0, 20 °C.

In the current paper, we describe examples of further gCuHCF application as a PO mimic in ABSs for the detection of alcohols, specifically methanol, and glycerol. These ABSs utilize alcohol oxidase (AO) and glycerol oxidase (GlycO) as biorecognition elements (Figure 5 and Figure 6), highlighting the versatility of gCuHCF in ABS development.

These modifications facilitate enhanced sensitivity in methanol detection through the catalytic and electro-mediator properties of the gCuHCF combined with the enzymatic activity of AO. The sensitivity of the AO/gCuHCF/GE for methanol is 342 A·M^−1^·m^−2^, which is five times higher than the sensitivity of the bi-enzyme ABS with the configuration AO/PO/GE (66 A·M^−1^·m^−2^) that we developed earlier [28]. The constructed AO-based ABS is stable during two weeks of storage at 4 °C in vapors over the 50 mM phosphate buffer, pH 7.0 (Figure S3).

The peculiarity of the just-developed mono-enzyme electrode for glycerol analysis is in its simplicity and cost-effectivity. This configuration ensures optimized catalytic activity and electron transfer, making the GlycO/gCuHCF/GE an effective amperometric biosensor for glycerol detection.

The main properties of the previously developed and novel gCuHCF-based ABSs are summarized in Table 1, which provides a comparison of their key characteristics, especially sensitivity, LOD, linear range, and K_M_^app^. These parameters highlight the versatility and effectiveness of gCuHCF as a platform for various biosensing applications.

As shown in Figure 6c and Table 1, the sensitivity of the ABS for glycerol is relatively low, specifically 17 A·M^−1^·m^2^.

However, the aim of this work was not to develop the most efficient ABSs but rather to present potential approaches for future experiments in the construction of novel, practically important mono-enzyme–nanozyme-based ABSs using gCiHCF.

It is worth mentioning that the key feature of the newly developed mono-enzyme electrodes for alcohol analysis lies in their simplicity and cost-effectiveness. By utilizing a single enzyme (AO or GlycO), in combination with the catalytically active and electro-mediating properties of gCuHCF, these ABSs eliminate the need for complex multi-enzyme systems or expensive reagents. This streamlined design reduces production costs and makes electrodes more accessible for various analytical and industrial applications.

To achieve enhanced analytical characteristics for such amperometric biosensors (ABSs), it is necessary to conduct numerous experiments focused on optimizing the procedures involved in ABS development and their working conditions. This issue has been discussed in detail in our previous papers [21,22,23,24].

There are several effective strategies to enhance the sensitivity of enzyme-based biosensors. The efficiency of electron transfer (ET) from the enzyme to the electrode can be improved by ensuring close contact between the enzyme and the electrode surface. A well-established approach to enhance ET effectiveness is the use of electroactive mediators—either freely diffusing or immobilized—coupled with the enzyme on the electrode surface. In our previous works, we demonstrated that electroactive PO-like nanozymes, including gCuHCF, serve a dual role as both immobilized mediators and artificial peroxidases, significantly enhancing the sensitivity of enzyme–nanozyme ABSs. For the optimization of the chemo-sensor and the construction of the biosensor, the quantity of gCuHCF material on the surface of the GE, as well as the enzyme/gCuHCF ratio, must be experimentally determined [21,22,23,24].

Another way to enhance the sensitivity of an ABS is to select the optimal amount of the enzyme carefully. Our previous results [21] highlighted the importance of using an optimal, rather than maximal, quantity of the enzyme. Increasing the enzyme concentration beyond the optimal level does not necessarily lead to higher sensitivity; instead, it may negatively impact the diffusion process within the protein-enriched recognition layer, ultimately reducing the ABS performance. Additionally, the optimal buffer, pH, and working potential must be determined experimentally to optimize the conditions for ABS usage. The data in Table S1 support these statements.

### 3.3. Other Analytical Capabilities of gCuHCF-Modified Electrode

Other examples of gCuHCF application are the following: gCuHCF is a platform for enzyme (Fc*b*_2_) concentration and stabilization, as well as a chemo-sensor on ammonium ions. To investigate the selectivity of gCuHCF, modified GE was tested for its ability to respond to various analytes, including glucose and other monosaccharides, primary alcohols, organic acids, ammonium ions, and others. Amperometric analysis was performed using cyclic voltammetry (CV) and chronoamperometry at different potentials (−50 mV and +150–200 mV) in various buffer solutions, with pH values ranging from 4.0 to 8.0. The gCuHCF/GE was found to produce negligible current responses to L-lactate and ammonium ions under the studied conditions [21].

**Figure 7:**
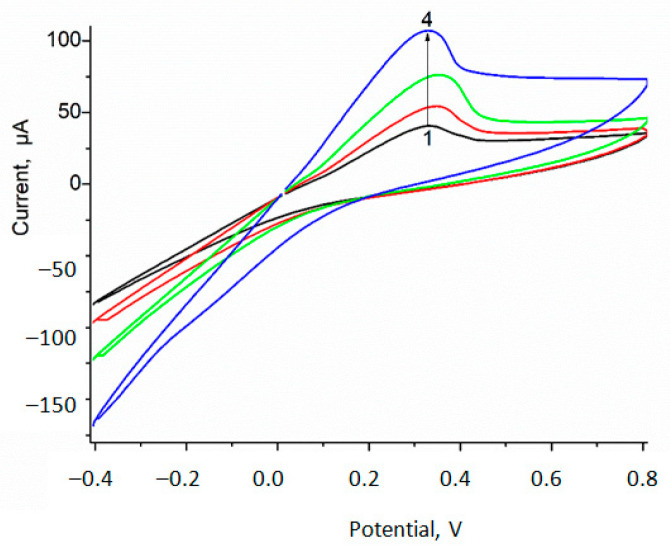
CV profiles of fresh-prepared gCuHCF/GE as biosensor on lactate: outputs on substrate addition up to concentration (mM): 0 (**1**, black), 15 (**2**, red), 30 (**3**, green), and 50 (**4**, blue). Conditions: scan rate 50 mV·s^−1^ vs. Ag/AgCl as reference electrode, 50 mM acetate buffer, pH 6.0, 20 °C. The sensing layer contains 4.1 mU Fc*b*_2_.

In this work, we demonstrated that Fc*b*_2_ was concentrated from the diluted solutions due to co-precipitation with gCuHCF-based micro-nanoflowers during their formation. gCuHCF being immobilized on GE may be ABS on lactate. CV analysis (Figure 7) proved that current output on lactate addition correlates with Fc*b*_2_ activity in the sensing layer.

We investigated the ability of gCuHCF/GE to detect lactate in a model solution. For this purpose, the CV analysis was performed in the optimal buffer for gCuHCF—50 mM sodium acetate (NaAc), pH 4.5. As shown in Figure 7, the CV profiles exhibited significant current responses to lactate in the anodic field at voltages of 200–400 mV and in the cathodic field at voltages of –400–0 mV.

**Figure 8:**
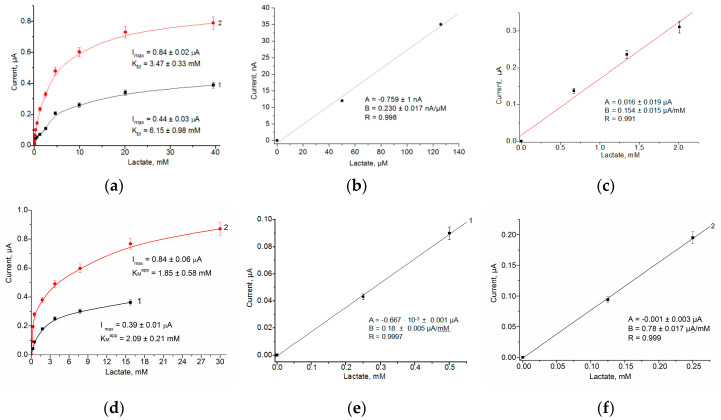
Characteristics of the gCuHCF/Ges as ABS-1 and ABS-2 for lactate: the dependence of the current response on increasing concentrations of the analyte is demonstrated in both wide (**a**,**d**) and linear ranges (**b**,**c**,**e**,**f**). ABSs contain different amounts of fresh gCuHCFs: 5 µL (**1**, black line) and 10 µL (**2**, red line). Measurements were performed in 50 mM acetate buffer, pH 6.0 (**a**–**c**), and in 50 mM phosphate buffer, pH 7.0 (**d**–**f**), working potential of +300 vs. Ag/AgCl (reference electrode), at 23 °C mV.

To further evaluate the potential of gCuHCF/GE as a (bio)sensor for lactate, a working potential of +300 mV was selected for detailed analysis. Figure 8 illustrates the effect of lactate addition on the constructed electrodes ABS-1 and ABS-2. The hNF composition, consisting of fresh gCuHCF on the GE surface, includes 2 U/mL of Fc*b*_2_. ABS-1 and ABS-2 incorporate 10 mU (Figure 8a,b,e) and 20 mU (Figure 8d,c,f) of the enzyme, respectively. Among these, ABS-2 demonstrated the highest sensitivity to lactate (62 A·M^−1^·m^−2^), which can be attributed to the greater quantity of gCuHCF on the GE surface and the optimal conditions for Fc*b*_2_ activity, specifically in 50 mM phosphate buffer at pH 7.0.

Currently, limited data are available on mono-enzymatic ABSs based on ammonia/ammonium-sensitive nanochelators—nanocomposites capable of forming redox-active coordination compounds with ammonia [31,32,33,34,35,36,37,38,39,40]. Recently, metal oxides in the form of nanoparticles or nanocomposites useful for the amperometric detection of ammonia have been described in detail in our publications. Furthermore, a hypothetical mechanism for the action of ammonium nanochelators in electron transfer reactions has been proposed [38]. Only a few studies focus on ABSs, based on metal oxides and sulfur-containing NPs as ammonia chemosensors. Thus, the construction of bioelectrodes incorporating enzymes conjugated with metallic ammonia-sensitive NPs in a bioselective layer remains a significant challenge.

**Figure 9:**
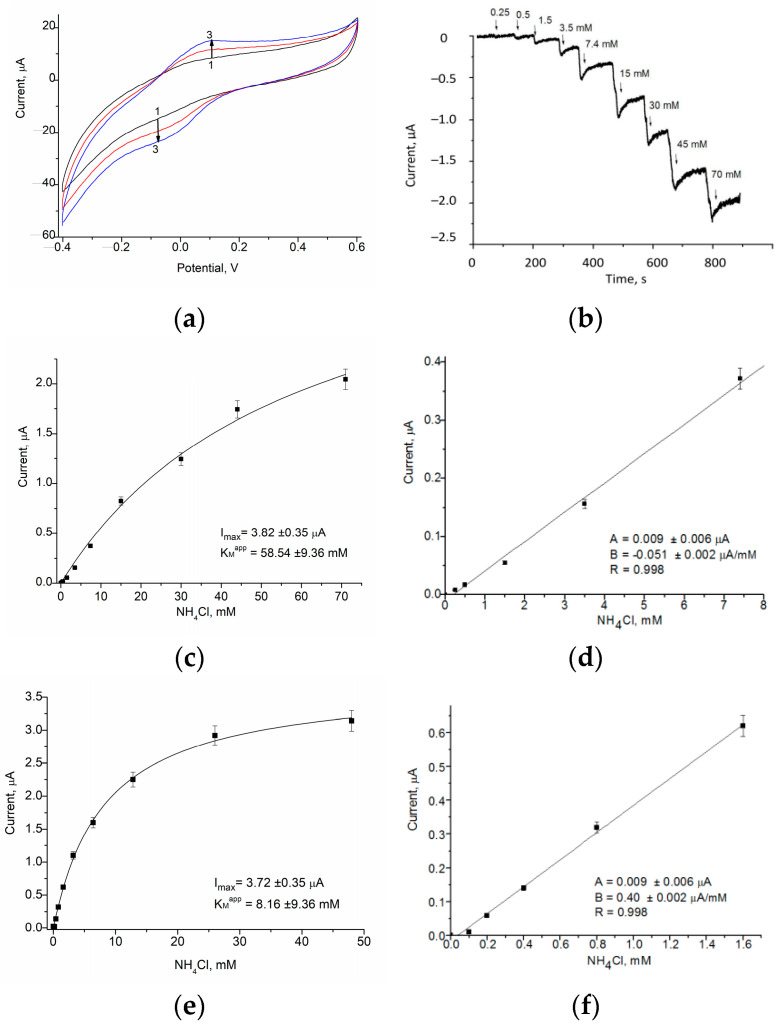
Characteristics of gCuHCF/GE as a chemosensor for ammonium ions: CV profiles as outputs on ammonium chloride addition (**a**) up to concentration (mM): 0 (**1**, black), 30 (**2**, red), and 60 (**3**, blue); chronoamperogram (**b**), dependence of the amperometric response on increasing ammonium chloride concentrations (**c**–**f**) in wide (**c**,**e**) and linear ranges (**d**,**f**). Conditions: scan rate (for **a**) is 50 mV·s^−1^ vs. Ag/AgCl (reference electrode); temperature: 23 °C. Working potentials: +150 mV (**b**,**c**,**d**) and −100 mV (**e**,**f**). Buffers: 50 mM sodium acetate, pH 4.5 (**b**–**d**) and 50 mM sodium phosphate, pH 8.0 (**e**,**f**).

Figure 9 illustrates the characteristics of gCuHCF/GE as sensors for the amperometric detection of ammonium ions. These findings demonstrate the potential application of gCuHCF/GE as a chemosensing platform in ABSs utilizing enzymes such as urease, arginine deiminase, arginine oxidase, and creatinine deiminase, which generate ammonia ions during the catalysis of their specific substrates.

According to the data in Figure 9, the key analytical parameters of gCuHCF/Ges as ammonium nanochelators depend on the buffer composition and pH, as well as on the working potential and the quantity of gCuHCF on the GE surface. The sensitivities of gCuHCF/GE to ammonium ions are 7.0 A·M^−1^·m^−2^ at pH 4.5 and 55 A·M^−1^·m^−2^ at pH 8.0. Although these values are significantly lower than those obtained using our synthesized copper nanoparticles [38], they are much higher than those reported for a comparable ammonium-sensitive sensor based on green-synthesized MnO_2_ nanoparticles [35].

### 3.4. gCuHCFs as Perovscites

**Figure 10:**
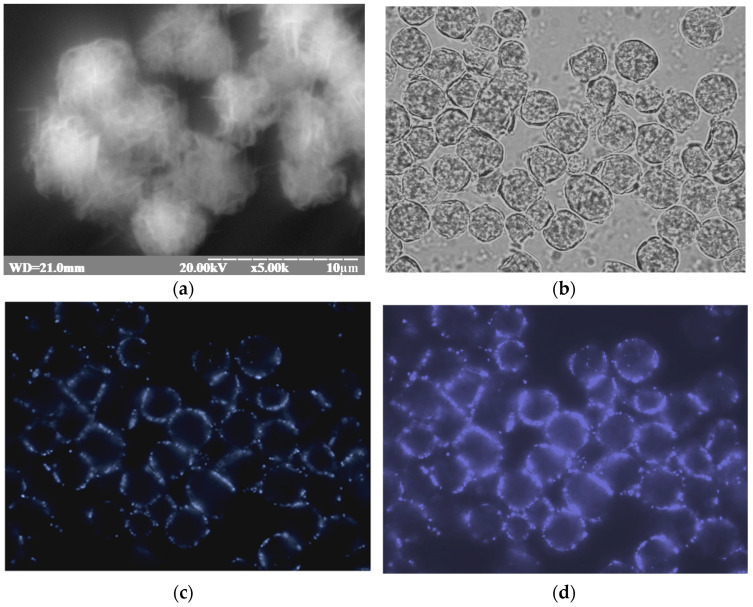

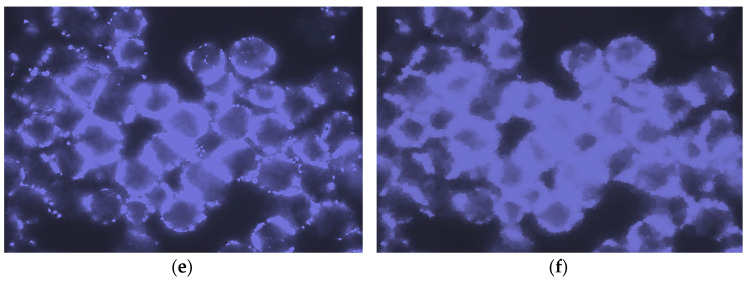
Microscopic characterization of freshly prepared gCuHCFs suspended in 50 mM phosphate buffer: SEM image (**a**) and fluorescence images (**b**–**f**) captured under different filters. The images include brightfield (**b**) and blue-light fluorescence (**c**–**f**), showing dynamic changes over 1 to 5 min after irradiation with a 100 ms exposure.

**Figure 11:**
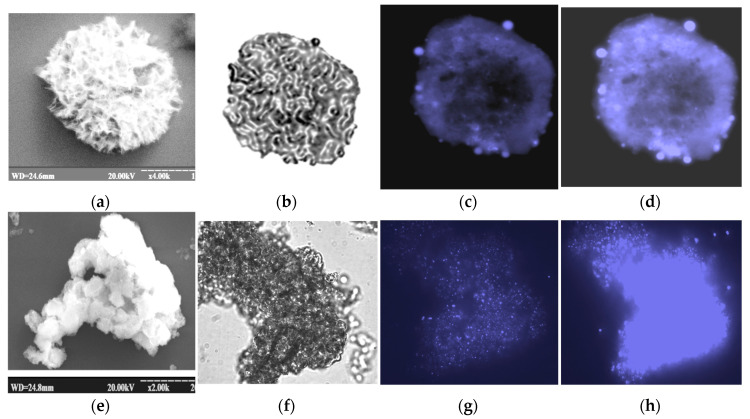
Microscopic characterization of gCuHCFs samples: SEM images (**a**,**e**) and fluorescence images (**b**–**d**,**f**–**h**) captured under different filters. The FM images include brightfield (**b**,**f**) and DAPI-stained fluorescence (**c**,**d**,**g**,**h**), showing dynamic changes over 1 to 5 min after irradiation with a 100 ms exposure. gCuHCF samples were kept for 1 month at 4 °C as a suspension in water (**a**–**d**) and as a lyophilized powder, which was suspended in water before characterization (**e**–**h**).

We studied the fluorescence properties of gCuHCFs and discovered their ability to enhance blue light emission. This finding demonstrates that gCuHCF exhibits perovskite-like characteristics. Figure 10 and Figure 11 illustrate the fluorescence emission of gCuHCFs under a DAPI filter, characterized by an excitation wavelength of 350 nm and an emission at 470 nm, confirming their ability to emit blue light. Figure S4 demonstrates the fluorescence spectra of the gCuHCF solution. A brief 3D screening study (Figure S4a) provides essential information for determining the conditions necessary for the detailed characterization of the gCuHCF. As a result, the emission and excitation spectra were recorded under optimal conditions (Figure S4b).

Metal–organic perovskites (MOPs) are a fascinating class of hybrid materials that combine the structural versatility of organic compounds with the robust physical properties of inorganic molecules [41,42,43,44,45,46]. MOPs share similarities with traditional inorganic perovskites, having a general formula of ABX_3_, where A is a larger organic or inorganic cation, B is a smaller metal cation, and X is typically a halide or oxide ion.

MOPs represent a promising platform for advancing technologies in energy, environment, and healthcare. The key properties of MOPs include structural versatility, exceptional electronic and optical characteristics, improved stability and processability, catalytic and electrocatalytic activity, biocompatibility, and suitability for sensing applications. MOPs are applied across diverse fields: in photovoltaics, particularly in perovskite solar cells (PSCs); in light-emitting devices due to their high luminescence efficiency and color purity; in (bio)sensors for environmental monitoring and clinical diagnostics; in supercapacitors, batteries, and fuel cells for energy storage and catalysis; and in reactors for absorbing or degrading toxic pollutants, particularly for water treatment and air purification [43,44].

Copper (Cu) is seen as an eco-friendlier alternative to traditional PSCs, which often use toxic lead (Pb). Cu can serve as a dopant in the perovskite structure, as a cation in the A-site of the ABX_3_ perovskite formula, replacing lead in compounds like CsCuCl_3_ or Cs_2_CuCl_4_, as a component of hole transport layers (HTLs) or charge-selective layers in PSC. Copper complexes are used as HTLs in PSC due to their high conductivity and cost-effectiveness, compared to precious metal-based HTLs (e.g., gold or platinum). Cu-containing 2D and 3D perovskites offer notable advantages, including higher thermal and UV light stability compared to their Pb-based counterparts, which are prone to degradation under environmental stressors [45,46]. However, Cu-based perovskites still present challenges, such as the complexity of their synthesis. Research trends in the development of PSCs are focusing on hybrid Cu-based perovskites that combine organic and inorganic components to achieve tunable properties. Another area of exploration involves integrating copper-based materials with tandem solar cells to enhance overall efficiency.

## 4. Conclusions

Copper-containing metal–organic nano- and micro-composites are promising catalysts for biosensing applications. In the current work, we demonstrate that gCuHCF, synthesized via Fc*b*_2_ and incorporated into a flower-like structure, can function as a biosensor for lactate. Furthermore, under specific conditions, gCuHCF produces moderate signals for ammonia ions, suggesting its potential use as a chemosensor for ammonia ions in ABSs based on deiminases or other enzymes producing ammonium ions as a byproduct of their enzymatic reactions. As a PO mimic, gCuHCF can also be utilized in electrochemical and optical (bio)sensors for detecting H_2_O_2_, either as a standalone analyte or as a byproduct of an oxidase-catalyzed reaction.

One of the most remarkable properties of gCuHCF, discovered for the first time in this study, is its ability to enhance fluorescence under blue-light irradiation, exhibiting characteristics akin to those of perovskites. It was reported that copper-containing PSCc are an emerging area of research in photovoltaic technology, aiming to address efficiency, non-toxicity, thermal and chemical stability, developing cost-effective and scalable synthesis methods for their commercial viability, and sustainability challenges in solar energy conversion. gCuHCF, exhibiting perovskite-like characteristics, holds potential as a cost-effective material for developing alternative energy devices, such as solar batteries.

Thus, the originality of gCuHCF lies in its multifunctionality, acting as a PO mimic, a chemo-sensor for ammonium ions, a biosensor for L-lactate, and in being a perovskite. Additionally, using gCuHCF as a PO-like NZ, novel oxidase-based sensors were developed, including an optical biosensor for L-arginine analysis and amperometric biosensors for methanol and glycerol determination. Looking ahead, hexacyanoferrates of transition metals, particularly gCuHCF synthesized via various enzymes, hold great potential as effective platforms for constructing multifunctional amperometric and optical (including fluorescent) (bio)sensors. However, significant research and development efforts are essential to address current limitations and fully harness the potential of these innovative materials for a wide range of applications.

## Figures and Tables

**Table 1:** Analytical characteristics of the oxidase- and gCuHCF-based biosensors.

Enzyme	GO	ArgO	Laccase	Fc*b*_2_	AO	GlycO
Substrate	glucose	L-arginine	cathechol	L-lactate	methanol	glycerol
Working potential, V	−0.25	−0.15	0.23	0.075	0.15	0.15
K_M_^app^, mM	0.35	0.12	2.03	1.90	0.47	9.99
LOD, μM	10	5	0.5	10	25	250
Linear range, up to mM	0.5	0.1	0.2	0.3	0.25	7.0
Sensitivity, A·M^−1^·m^−2^	710	573	762	80	342	17
Reference	[21]	[22]	[23]	[24]	This paper	This paper

## Data Availability

The data are available in this publication.

## Supplementary Materials

The supporting information can be downloaded at https://www.mdpi.com/article/10.3390/bios15030157/s1. The supporting information includes details on the properties of gCuHCF, such as its structural and compositional characteristics (Figure S1), selectivity tests (Figure S2), stability of the AO/gCuHCF/GE biosensor for methanol determination (Figure S3), fluorescence spectra of the gCuHCF solution (Figure S4), and analytical characteristics of the previously developed GO/gCuHCF/GEs for glucose analysis (Table S1).

## Abbreviations

ABS	Amperometric biosensor
ABTS	2,2′-azinobis (3-ethylbenzothiazoline-6-sulfonate) diammonium salt
AO	Alcohol oxidase
ArgO	Arginine oxidase
DLS	Dynamic Light Scattering
Fc*b*_2_	Flavocytochrome *b*_2_
FM	Fluorescent microscopy
FTIR	Fourier transform infrared spectroscopy
GE	Graphite electrode
gHCF	Hexacyanoferrates obtained via green synthesis using enzyme
GlycO	Glycerol oxidase
GO	Glucose oxidase
HTLs	Hole transport layers
*K_M_^app^*	Michaelis–Menten constant, apparent
NPs	Nanoparticles
NZs	Nanozymes
o-DZ	o-dianisidine
PO	Peroxidase
PSC	Perovskite solar cells
SEM-XRM	Scanning electron microscopy coupled with X-ray microanalysis
XRD	X-ray diffraction method
