# Supplementary material for: Copper Hexacyanoferrates Obtained via Flavocytochrome b2 Assistance: Characterization and Application
Source: Biosensors (Basel). 2025 Mar 2;15(3):157. doi: 10.3390/bios15030157 (PMC11940147; doi:10.3390/bios15030157)

Supporting information

**Copper hexacyanoferrates obtained via flavocytochrome *b*_2_ assistance: characterization and application**

**Galina Gayda ^1,^*, Olha Demkiv ^1^, Nataliya Stasyuk ^1^, Halyna Klepach ^2^, Roman Serkiz ^1^, Faina Nakonechny ^3^ Mykhailo Gonchar ^1^ and Marina Nisnevitch ^3,^***

| **Citation:** To be added by editorial staff during production.  Received: date  Revised: date  Accepted: date  Published: date  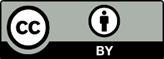  **Copyright:** © 2024 by the authors. Submitted for possible open access publication under the terms and conditions of the Creative Commons Attribution (CC BY) license (https://creativecommons.org/licenses/by/4.0/). |
| --- |

^1^ Department of Analytical Biotechnology, Institute of Cell Biology National Academy of Sciences of Ukraine,

14/16 Drahomanov Str., 79005 Lviv, Ukraine; galina.gayda@nas.gov.ua (G.G.); demkivo@nas.gov.ua (O.D.); stasukne@nas.gov.ua (N.S.); rserkiz@gmail.com (R.S.); gonchar@cellbiol.lviv.ua (M.G.)

^2^ Department of Biology and Natural Sciences, Drohobych Ivan Franko State Pedagogical University, 82100 Drohobych, Ukraine; pavlishko@yahoo.com

^3^ Department of Chemical Engineering, Ariel University, Kyriat-ha-Mada, Ariel 4070000, Israel; fainan@ariel.ac.il (F.N.); marinan@ariel.ac.il (M.N.)

***** Correspondence: galina.gayda@nas.gov.ua or galina.gayda@gmail.com (G.G.); marinan@ariel.ac.il (M.N.)


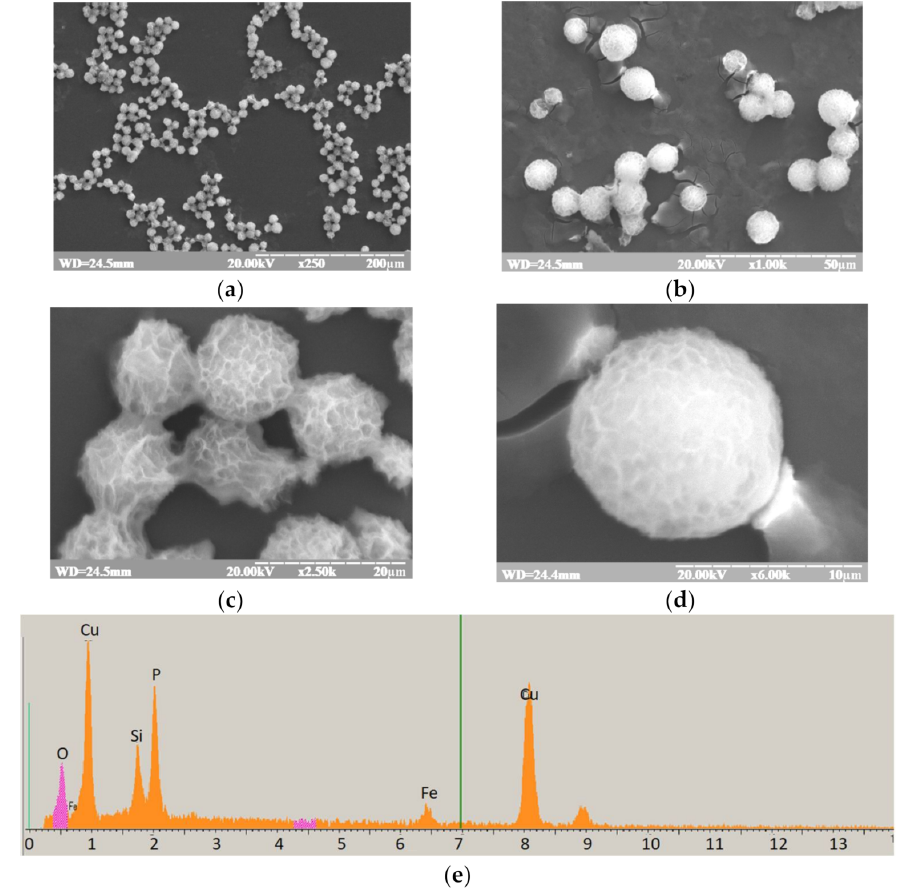


**Figure S1**. The results of gCuHCF study using SEM with RSM: (a–d)—SEM images at different magnifications; (e)—X‐ray spectral characteristics [reprinted from our paper 21].

**
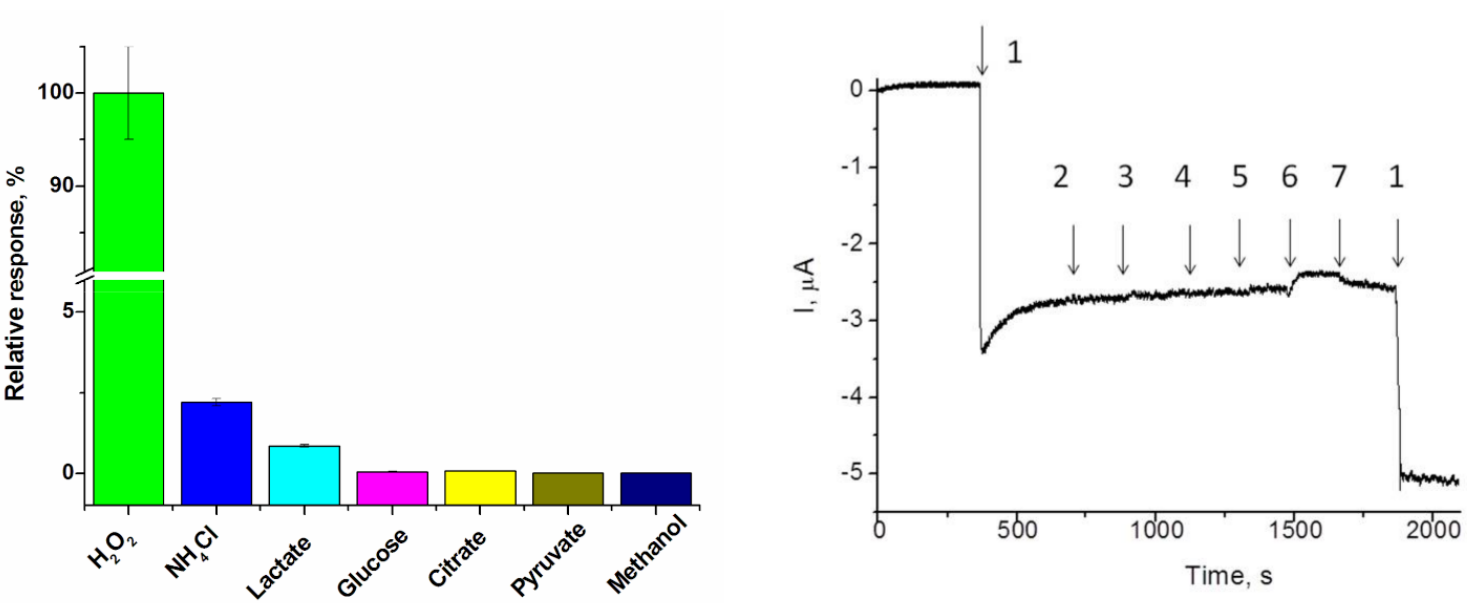
**

1. (**b**)

**Figure S2**. The selectivity tests for gCuHCF/GE: (**a**)—current responses in relative units (%), on the

added analytes up to 2 mM concentration, as a ratio of the detected signals to the value of the highest

current response; (**b**)—chronoamperograms as outputs on the added analytes (1–7) up to 0.5 mM

concentration: (1)—H_2_O_2_, (2)—glucose, (3)—glycerol, (4)—methanol, (5)—sodium citrate, (6)— sodium

lactate, (7)—ammonium chloride. Conditions: working potential −50 mV vs. Ag/AgCl (refer‐

ence electrode), 50 mM NaOAc buffer, рН 4.5 at 23 °C [reprinted from our paper 21].


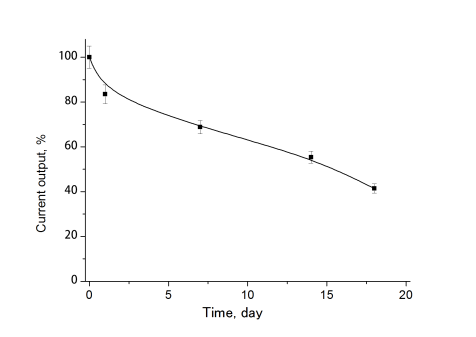


**Figure S3**. Stability of the AO/gCuHCF/GE biosensor for the determination of 1 mM methanol.


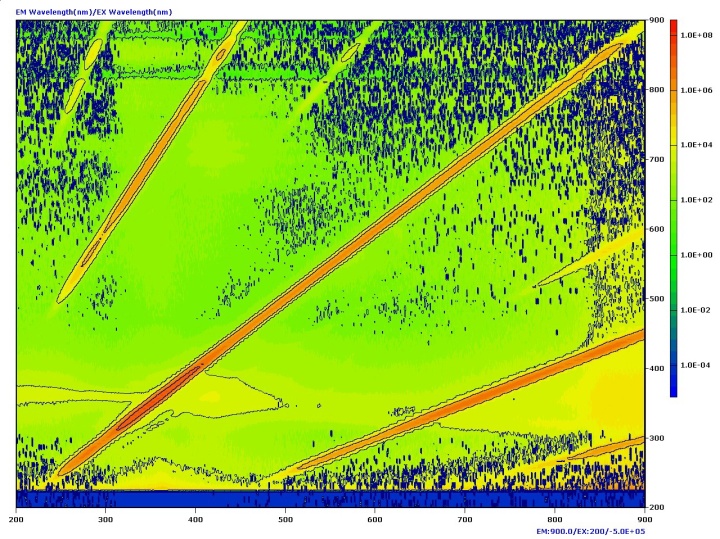

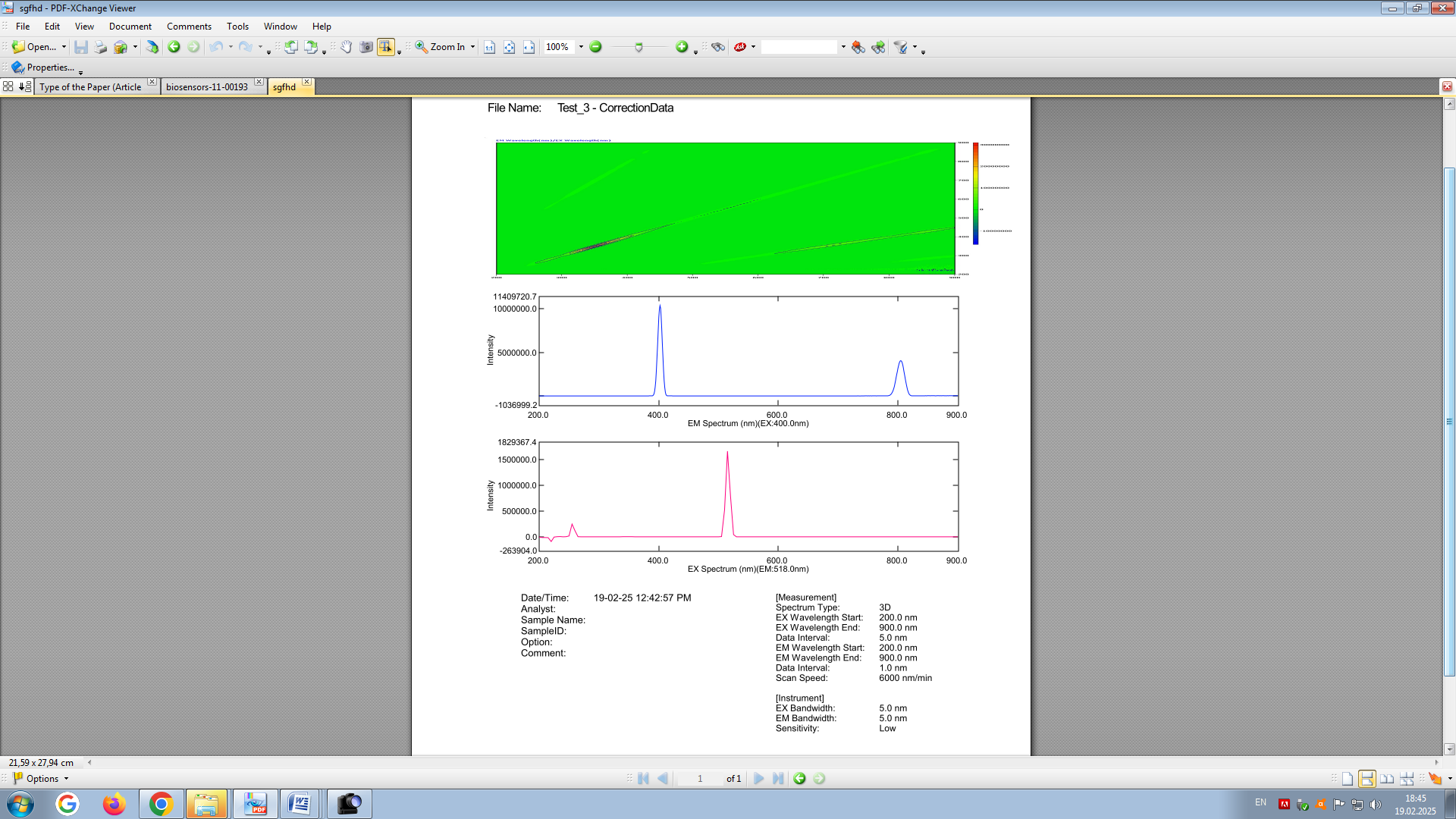


(**a**) (**b**)

**Figure S4**. Fluorescence spectra of the gCuHCF solution at a concentration of 3.5 mg/mL. (**a**) – A brief 3D screening study; (**b**) – Emission (top) and excitation (bottom) spectra obtained under the optimal conditions.

**Table S1**. Analytical characteristics of the developed GO/gCuHCF/GEs for glucose analysis [reprinted from our paper 21].


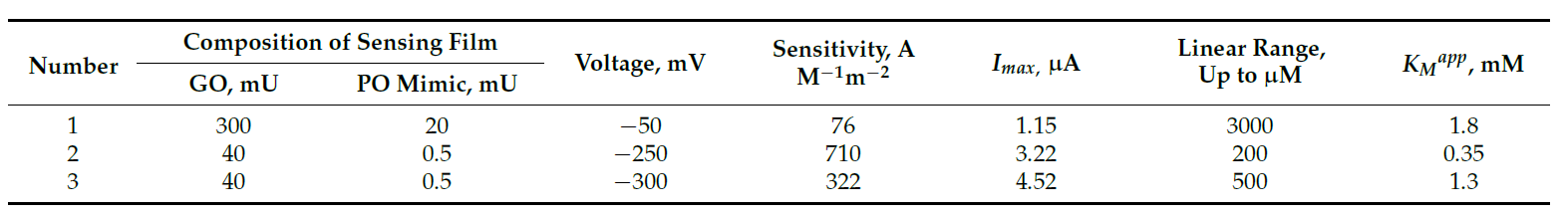

Supplement: Supplementary file 1 [file biosensors-15-00157-s001.zip › biosensors-3446669-supplementary.docx]
